# Breastfeeding in public: “You can do it?”

**DOI:** 10.1186/s13006-014-0026-1

**Published:** 2014-12-20

**Authors:** Lisa H Amir

**Affiliations:** Judith Lumley Centre, La Trobe University, 215 Franklin St, Melbourne, VIC 3000 Australia

**Keywords:** Breastfeeding in public, Public perception, Determinants of breastfeeding

## Abstract

On a regular basis there is an outcry about a mother who has been told to cover up or move away from a public area while she is breastfeeding. Mothers should feel free to breastfeed whenever they need to. However, the increasing market for “nursing covers” to hide the breast while feeding is evidence of changing perceptions. Discomfort with the idea of breastfeeding in public has been cited as a reason for some women choosing not to initiate breastfeeding or planning a shorter duration of breastfeeding. Other women are choosing to express and bottle-feed their expressed milk when they are in public. In many cultures today there is a conflict between the concept of breast milk being pure (like tears), and contaminated or “dirty” (like genital secretions or vomit). In these settings the female breast may be considered primarily a sexual organ, and therefore a private part of the body, which needs to be invisible in the public arena. In order to increase breastfeeding initiation and duration and to reduce health inequities breastfeeding needs to be more visible. Let’s strive together to make breastfeeding in public unremarkable.

## Commentary

**Figure 1:**
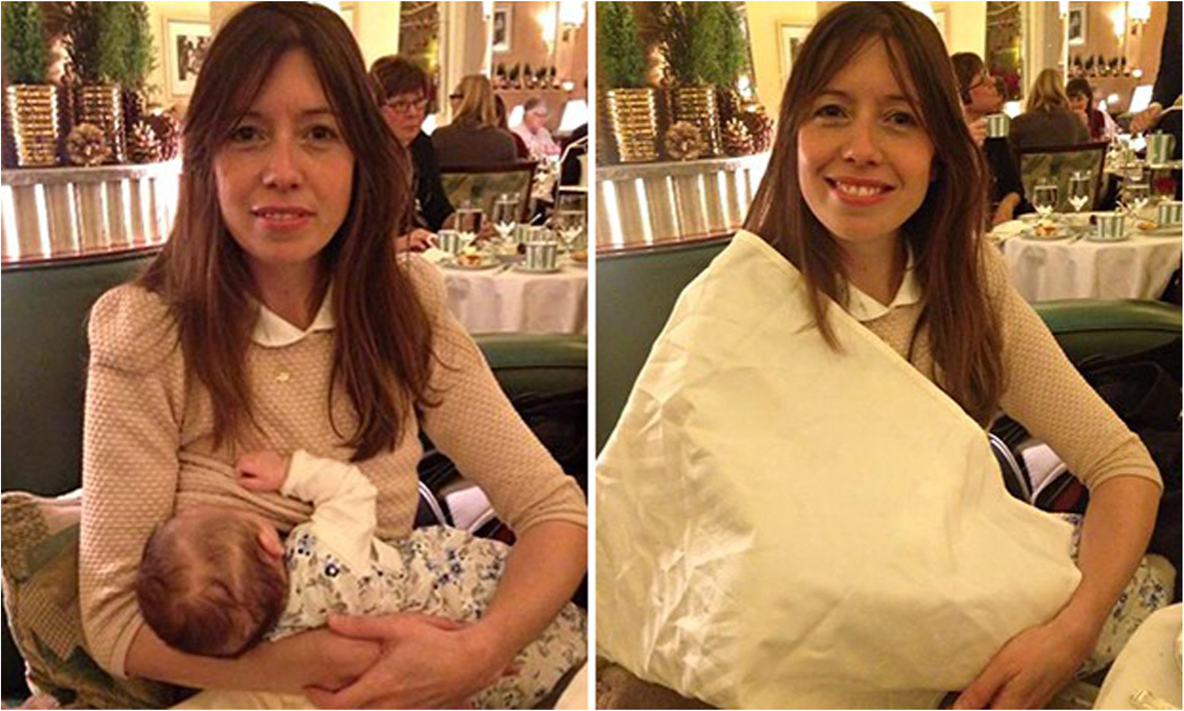
**Louise Burns at Claridge’s hotel, London, UK (1 December 2014, via Twitter).**

Babies need to feed frequently; human milk is low in fat and similar in content to other mammals who feed their young at short intervals. Therefore, mothers with children breastfeed as they go about their daily activities. Sounds simple. However, on a regular basis there is an outcry about a mother who has been told to cover up or move away from a public area while she is breastfeeding. This week, a mother in an up-market London hotel was told to cover herself with a large serviette when feeding her 12 week old baby, as shown in Figure 1 [1]. In August 2014 in the USA, a woman was asked to move to the bathroom because she was breastfeeding her six-week-old baby during a shopping trip [2]. In one well-known incident an Australian television host commented that he thought it was “fair enough” that an attendant had asked a mother to be more discreet while breastfeeding at a public pool [3].

Mothers should feel free to breastfeed whenever they need to. However, the increasing market for “nursing covers” [4] to hide the breast while feeding is evidence of changing perceptions. When I was a breastfeeding mother in the 1980s in Australia, there was no talk about “breastfeeding discreetly”, and I was shocked when I attended my first breastfeeding conference in the USA in 1991 and saw a fellow conference attendee cover her baby’s face while breastfeeding at the lunch table. Since then, many products have been developed and marketed to enable women to feel breastfeeding can be acceptable while in the public sphere. The marketing strategy for one company is to advertise that their product “Eradicates any embarrassment issues for the mother and members of the public” [5].

Covering the breasts during feeding has implications for maternal and infant health and well-being. In my clinical practice, I’ve seen a woman who developed mastitis after feeding awkwardly because she was concealing her breast in a public setting. I feel saddened whenever I see this: where is the eye contact and reciprocal communication between mother and child?

In Australia, and many other countries, the right to breastfeed in public has been established by law. Law makers have acknowledged that the right to food is a fundamental human right [6]. However, the general public may not be aware of this, and prudishness about seeing a baby at the breast can lead to waiters, security guards, shop attendants and others responding inappropriately when the act of breastfeeding occurs in “their space” [7].

Families may feel more comfortable in public spaces such as parks and gardens than in places like shopping centres. While food courts in shopping malls can be convenient for families when they are out of the home, they are often busy, noisy places and new mothers can find the lack of a quiet corner intimidating. Mothers report that that they felt more comfortable breastfeeding in a park than a shopping mall [8]. Particularly, women felt supported breastfeeding in a group situation in a park; they feel less comfortable breastfeeding alone in a park [9].

Discomfort with the idea of breastfeeding in public has been cited as a reason for some women choosing not to initiate breastfeeding [10] or planning a shorter duration of breastfeeding [11]. Other women are choosing to bottle -feed their expressed milk when they are outside the home [12]. Although health authorities around the world promote breastfeeding, in practice the image of the infant bottle is ubiquitous and still often seen as the normal way to feed a baby. The increasing practice of expressing milk for healthy term infants might help women extend their duration of breast milk feeding [13], but it doesn’t help normalise breastfeeding at the breast.

In many cultures today there is a conflict between the concept of breast milk being pure (like tears), and contaminated or “dirty” (like genital secretions or vomit) [9]. Women may feel ashamed of leaking breasts if milk is considered a bodily fluid like urine or menstrual blood that needs to be kept hidden from sight and controlled [9]. Many cultures consider the female breast primarily as a sexual organ, and therefore a private part of the body, which needs to be invisible in the public arena (yet they have no similar objection to breasts and cleavages being displayed for other purposes) [14].

Anxiety about breastfeeding in front of other people particularly affects breastfeeding duration in women with low self-confidence or who feel society disapproves of breastfeeding in public [12]. Research has demonstrated this fear of breastfeeding in public in young women, low income women, and immigrant women in western countries [15-17].

The public health message that breastfeeding is important for maternal and child health is not enough. Groleau and colleagues point to “the urgent need for reintroducing the nutritional role of the breast into various social and public spaces including the medias. Reintroducing the normality of breastfeeding in visible public places through images and pictures of women of all ages, body types and styles would be a positive step toward making breastfeeding an infant-feeding *habitus* – thus morally acceptable – in western countries as opposed to a sexually provocative practice” [17] p. 258.

In order to increase breastfeeding initiation and duration and reduce health inequities breastfeeding needs to be more visible. Can we have a middle way between the closed- off breastfeeding or “lactation” room and the bustling open food court? We need to work with communities to determine the most appropriate means of doing this. Do billboards or posters on buses work? The New Zealand Ministry of Health released the “Lucy poster” of the actor Lucy Lawless and her child as part of World Breastfeeding Week in August 2002. The poster – titled *“Breastfeeding – my best role ever”* – made breastfeeding visible and emphasised that breastfeeding is work that women do, yet was controversial at the time [18]. Cardboard cut-outs of women breastfeeding have been used in the UK and the USA [9]. Many communities have conducted breastfeeding in the park events, breastfeeding en masse events, or breastfeeding sit-ins. In Montreal, Canada, a group put together a YouTube video declaring “Nursing is normal” [19]. Research is needed to evaluate these and other strategies in order to normalise the act of breastfeeding in public.

**Figure 2:**
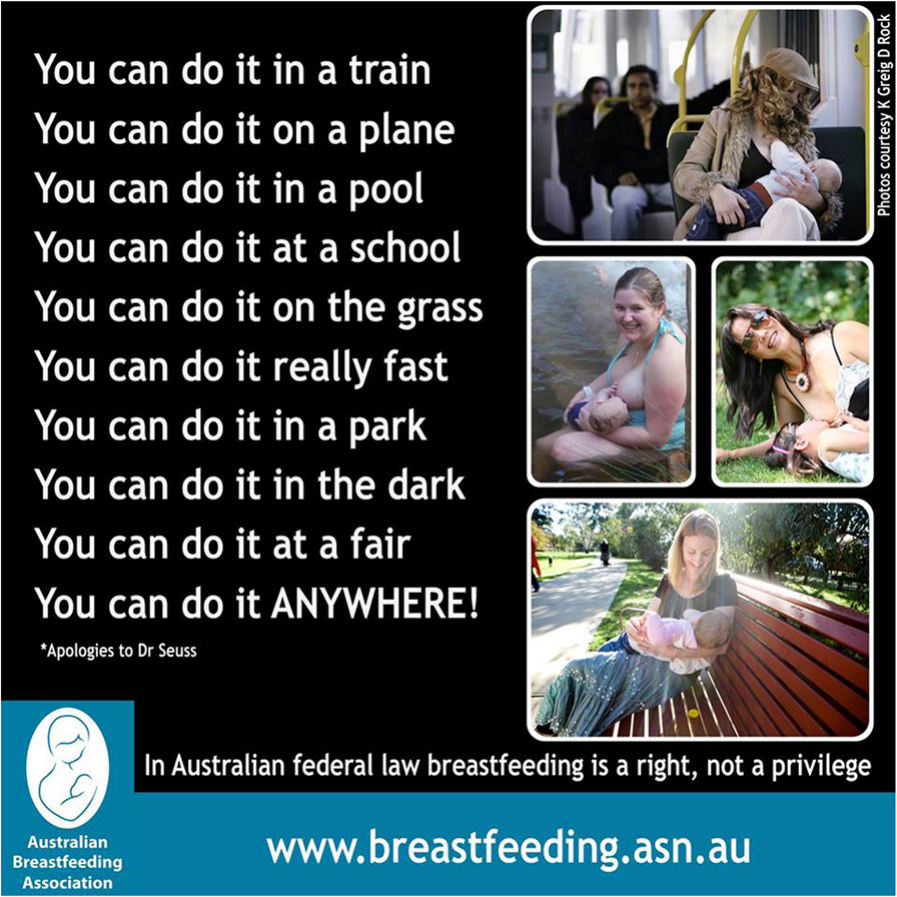
**Australian Breastfeeding Association poster: “You can do it . . .” (used with permission).**

Let’s strive together to make breastfeeding in public unremarkable. As the Australian Breastfeeding Association poster says “You can do it on a train, you can do it on a plane . . .” (Figure 2, with apologies to Dr Seuss).

## Consent

Written informed consent was obtained from the individual for the publication of the images published on Twitter.
